# Comparison of alternative versions of the job demand-control scales in 17 European cohort studies: the IPD-Work consortium

**DOI:** 10.1186/1471-2458-12-62

**Published:** 2012-01-20

**Authors:** Eleonor I Fransson, Solja T Nyberg, Katriina Heikkilä, Lars Alfredsson, De Dirk Bacquer, G David Batty, Sébastien Bonenfant, Annalisa Casini, Els Clays, Marcel Goldberg, France Kittel, Markku Koskenvuo, Anders Knutsson, Constanze Leineweber, Linda L Magnusson Hanson, Maria Nordin, Archana Singh-Manoux, Sakari Suominen, Jussi Vahtera, Peter Westerholm, Hugo Westerlund, Marie Zins, Töres Theorell, Mika Kivimäki

**Affiliations:** 1 Institute of Environmental Medicine, Karolinska Institutet, Box 210, SE-171 77 Stockholm, Sweden; 2 School of Health Sciences, Jönköping University, Jönköping, Sweden; 3 Finnish Institute of Occupational Health, Helsinki, Finland; 4 Department of Public Health, Ghent University, Ghent, Belgium; 5 Department of Epidemiology and Public Health, University College London, London, UK; 6 MRC Centre for Cognitive Ageing and Cognitive Epidemiology, University of Edinburgh, Edinburgh, UK; 7 Versailles-Saint Quentin University, Versailles, France; 8 School of Public Health, Université Libre de Bruxelles, Brussels, Belgium; 9 Inserm U1018, Centre for Research in Epidemiology and Population Health, Villejuif, France; 10 Department of Public Health, University of Helsinki, Helsinki, Finland; 11 Department of Health Sciences, Mid Sweden University, Sundsvall, Sweden; 12 Stress Research Institute, Stockholm University, Stockholm, Sweden; 13 Department of Public Health and Clinical Medicine, Occupational and Environmental Medicine, Umeå University, Umeå, Sweden; 14 Department of Public Health, University of Turku, Turku, Finland; 15 Folkhälsan Research Center, Helsinki, Finland; 16 Finnish Institute of Occupational Health, Turku, Finland; 17 Occupational and Environmental Medicine, Uppsala University, Uppsala, Sweden; 18 Department of Behavioral Sciences, University of Helsinki, Helsinki, Finland

**Keywords:** Job demands, Job control, Job strain, Work stress, Agreement

## Abstract

**Background:**

Job strain (i.e., high job demands combined with low job control) is a frequently used indicator of harmful work stress, but studies have often used partial versions of the complete multi-item job demands and control scales. Understanding whether the different instruments assess the same underlying concepts has crucial implications for the interpretation of findings across studies, harmonisation of multi-cohort data for pooled analyses, and design of future studies. As part of the 'IPD-Work' (Individual-participant-data meta-analysis in working populations) consortium, we compared different versions of the demands and control scales available in 17 European cohort studies.

**Methods:**

Six of the 17 studies had information on the complete scales and 11 on partial scales. Here, we analyse individual level data from 70 751 participants of the studies which had complete scales (5 demand items, 6 job control items).

**Results:**

We found high Pearson correlation coefficients between complete scales of job demands and control relative to scales with at least three items (r > 0.90) and for partial scales with two items only (r = 0.76-0.88). In comparison with scores from the complete scales, the agreement between job strain definitions was very good when only one item was missing in either the demands or the control scale (kappa > 0.80); good for job strain assessed with three demand items and all six control items (kappa > 0.68) and moderate to good when items were missing from both scales (kappa = 0.54-0.76). The sensitivity was > 0.80 when only one item was missing from either scale, decreasing when several items were missing in one or both job strain subscales.

**Conclusions:**

Partial job demand and job control scales with at least half of the items of the complete scales, and job strain indices based on one complete and one partial scale, seemed to assess the same underlying concepts as the complete survey instruments.

## Background

Work-related psychosocial factors or "stressors at work" have been linked to increased risk of ill health and mortality in some [1-8] but not all studies [9-12]. The reasons behind these inconsistencies may include differences in the socio-demographic characteristics of the study populations, variations in the stability of the work stressors during the follow-up, selection bias, and imprecise measurement, particularly of the exposure [13,14]. Surprisingly little attention has been paid to the fact that the operationalisation of work-related stressors have varied between cohort studies.

A frequently used measure of work stress is the two-dimensional job strain model, originally described by Karasek [15] and further developed, both empirically and psychometrically, by Karasek and Theorell [16]. This model postulates that jobs characterised by a combination of high psychological demands and low control (or low decision latitude), that is, job strain, are likely to elicit work-related psychosocial stress. High psychological demands in the workplace mean that the employee has to work intensively or rapidly, and may experience conflicting expectations. Job control, in turn, refers to the degree of decision-making authority (for example, having an influence on what task to do and how to carry it out) and skill discretion (e.g., the use of personal skills on the job).

The two standardised, widely used questionnaires developed to measure the demand and control dimensions, and hence job strain, are: the Job Content Questionnaire (JCQ) [17] and the Demand Control Questionnaire (DCQ) [18,19]. The number of demand (five in both the JCQ and DCQ) and control items (nine in the JCQ and six in the DCQ) vary somewhat, as do the enquiries, and their response scales differ. Most of the JCQ items are expressed as statements and the respondents are asked to report if they agree or disagree with the statement on a four level Likert scale, while DCQ items are expressed as questions with the response options being frequency based (e.g., "Do you have to work very fast?" - Often, sometimes, seldom, never).

Investigators examining the relationship between job strain and health outcomes have often used partial versions of the questionnaires. Study-specific questionnaires have also been developed which can differ from the JCQ or DCQ in terms of the number of items, content and wording of the questions, and response alternatives. It is important to understand whether the different survey instruments assess the same underlying concepts, as this has implications for the interpretation of findings across studies, harmonisation of multi-cohort data for pooled analyses, and design of future studies [20].

Accordingly, the aim of the present analyses was to evaluate the comparability of alternative job demands, job control and job strain measures by assessing agreement against the complete scale. To do so, we use data from six studies together with information from additional 11 European cohort studies that comprise the "Individual-participant-data meta-analysis in working populations" (IPD-Work) Consortium.

## Methods

### Study population

This study is part of the "Individual-participant-data meta-analysis in working populations" (IPD-Work) consortium of European cohort studies. This collaboration was established at a workshop, the annual Four Centers Meeting in London, in November, 2008. New cohort studies have subsequently been added. The overall aim of the IPD-Work consortium is to aggregate raw data from a series of studies in order to obtain reliable estimates of the influence of psychosocial risk factors at work on chronic diseases, mental health, disability, and mortality.

In IPD-Work, a pre-defined two-stage data acquisition protocol is being used. The first stage involves the acquisition of baseline data on work stress as well as socio-demographic and lifestyle factors and the definition and harmonisation of these baseline characteristics across the studies. The second stage involves the acquisition of data on disease outcomes ascertained subsequent to the baseline survey. The present analyses were based on stage one only and were thus conducted before any linkage to disease data, planned for the second stage.

We examined the agreement between complete and partial psychological demands and control scales separately in the six cohort studies of the IPD-Work consortium that had the complete job demands and job control scales. These were: the Job Stress Study I (Belstress, Belgium) [21]; the Gaz et Electricité Cohort Study (GAZEL, France) [22]; the Health and Social Support in Finland Study (HeSSup, Finland) [23]; the Swedish Longitudinal Occupational Survey of Health (SLOSH, Sweden) [24,25]; the Work, Lipids and Fibrinogen Study Norrland (WOLF N, Sweden) [26]; and the Work, Lipids and Fibrinogen Study Stockholm (WOLF S, Sweden) [26]. This resulted in an analytic sample of 70 751 participants. The IPD-Work cohort studies where only partial scales were available were: the Copenhagen Psychosocial Questionnaire Study, Denmark [27]; Danish Work Environment Cohort Study, Denmark [28]; Still Working study, Finland [29]; Finnish Public Sector study, Finland [30]; Heinz Nixdorf Recall Study, Germany [31]; Intervention Project on Absence and Well-being Study, Denmark [32]; KORA-Cooperative Health Research in the Region Augsburg/MONICA, Germany [33]; Netherlands Working Condition Survey, the Netherlands [34]; Permanent Onderzoek Leefsituatie/Continuous Survey on Living Conditions, the Netherlands [35]; PUMA-Copenhagen Burnout Inventory Study, Denmark [36]; and Whitehall II study, the United Kingdom [37]. We used the information on available job demands and control items in these 11 cohort studies to identify partial scales relevant to compare with the complete scales. The details of the design and participants in all 17 IPD-Work studies have been published previously [21-37] and are briefly described in Additional file 1.

The studies from which data was used in the present analysis were approved by the ethics committees of the University Hospital of Ghent and the Faculty of Medicine of the Université Libre de Bruxelles (Belstress); the National Commission Overseeing Ethical Data Collection in France (Commission Nationale Informatique et Liberté) (GAZEL); the Turku University Central Hospital Ethics Committee (HeSSup); the Regional Research Ethics Board in Stockholm (SLOSH); and the ethics committee at Karolinska Institutet, Stockholm (WOLF N and WOLF S). All studies adhered to the Helsinki Declaration on ethical principles for medical research involving human subjects. Details of ethical approval of all the IPD-Work studies are described in Additional file 1.

### Definition of the complete and partial scales of job demands and control

Complete scale measures of job demands and job control were based on five items from the psychological demands scales and six items from the control scales from the JCQ and DCQ (referred to hereafter as the "complete scales" - see table 1). This represented our referent. We omitted the three additional control items in the JCQ that did not have a corresponding item in the DCQ as a means of improving the harmonisation of the control scale across studies. We constructed partial scales based on the JCQ/DCQ items that were available in each of the IPD-work studies that did not have the complete scales. This resulted in a total of six partial demand scales; five partial control scales; and 10 partial job strain scales. The individual questionnaire items available in each study are presented in table 2.

**Table 1:** Items from the Job Content Questionnaire (JCQ) and Demand Control Questionnaire (DCQ) included in the IPD-Work

JCQ	DCQ
**Psychological demand**	**Psychological demand**

working very fast	work very fast

working very hard	work very intensively

no excessive amount of work	too much effort

enough time	enough time

conflicting demands	conflicting demands

**Control**	**Control**

learn new things	learn new things

high level of skill	high level of skill or expertise

require you to be creative	require you to take the initiative

repetitive work	same thing to do over and over again

a lot of say	deciding what you do at work

little decision freedom	deciding how you do your work

**Table 2:** Job demand and control items in the cohorts included in the IPD-Work meta-analysis (questionnaire used in study)*

	Belstress (JCQ), GAZEL (JCQ), HeSSup (JCQ), SLOSH (DCQ), WOLF N (DCQ), WOLF S (DCQ)	COPSOQ (Mainly DCQ)	DWECS (Mainly DCQ)	FPS (JCQ)	HNR (JCQ)	IPAW (DCQ)	KORA (Mainly JCQ)	NWCS (Other)	POLS (Other)	PUMA (Mainly DCQ)	Still Working (Other)	WH II (Mainly DCQ)
**Job demand**												

1. Working very fast	X	X	X		X	X	X	X	X	X		X

2. Working very hard/intensively	X			X	X		X	X				X

3. No excessive amount of work/too much effort	X			X	X		X	X				

4. Enough time	X	X	X	X	X	X	X	X	X	X	X	X

5. Conflicting demands	X	X	X				X			X	X	X

**Job control**												

1. Learn new things	X	X	X	X	X	X	X		X	X	X	X

2. High level of skill	X	X	X	X	X	X	X			X	X	X

3. Creativity/initiative	X	X	X	X	X	X	X	X	X	X		X

4. Repetitive work	X	X	X	X	X	X	X		X	X	X	X

5. A lot of say/what to do	X	X	X	X	X	X			X	X	X	X

6. Little freedom/how to do	X	X		X	X	X	X	X	X		X	X

Belstress-Belgian Job Stress Study I, Belgium; COPSOQ-Copenhagen Psychosocial Questionnaire Study, Denmark; DWECS-Danish Work Environment Cohort Study, Denmark; FPS-Finnish Public Sector Study, Finland; GAZEL-Gaz et Electricité Cohort Study, France; HeSSup-Health and Social Support Study, Finland; HNR-Heinz Nixdorf Recall Study, Germany; IPAW-Intervention Project on Absence and Well-being Study, Denmark; KORA-Cooperative Health Research in the Region Augsburg/MONICA, Germany; NWCS-Netherlands Working Condition Survey, Netherland; POLS-Permanent Onderzoek Leefsituatie/Continuous Survey on Living Conditions, Netherland; PUMA-Copenhagen Burnout Inventory Study, Denmark; SLOSH-Swedish Longitudinal Occupational Survey of Health, Sweden; Still Working-Still Working study, Finland; WH II-Whitehall II study, United Kingdom; WOLF N-Work, Lipids and Fibrinogen Study, Norrland, Sweden; WOLF S-Work, Lipids and Fibrinogen Study, Stockholm, Sweden.

* JCQ = Job Content questionnaire; DCQ = Demand control questionnaire; Mainly DCQ/Mainly JCQ = Minor modifications from the original questionnaire; Other = job strain scale with proxy items. For formulations of the items used in respective study, please contact the corresponding author.

All studies included in the IPD-Work Consortium were designed and initiated before the IPD-Work Consortium began; the choice of instrument to measure job strain therefore varies between studies. In some studies, the wording of the job strain items differed from those in the original JCQ or DCQ, but was judged by the study coordinating authors (EF, SN, KH, TT and MiK) to sufficiently resemble the original questions such that they could be used as proxy items. For example, the question on conflicting demands in Still Working study was expressed as "Do your superiors or workmates give you contradictory orders or instructions?" as compared with the corresponding item in the DCQ "Does your work often involve conflicting demands?"; and in POLS the item "Do you have to work under great time pressure?" was judged to capture the same content as "Do you have enough time to do everything?" in the DCQ. The scales with proxy items are labelled as "other" in table 2. Some scales were very similar to the JCQ or DCQ and only differed from them in minor aspects; they are labelled as "mainly JCQ" or "mainly DCQ" in table 2.

Using our analytical sample from the six studies with the complete scales, mean response scores for job demands items and for job control items were calculated for each study participant, for both the complete and the different partial scales. For each scale, the mean response score was calculated for participants who had answered half or more of the demand or control questions in that specific scale. However, where only two items were used in a partial scale, both items had to have non-missing values for the mean score to be calculated.

The presence of job strain was defined as having high demands (i.e., higher than the study-specific median of the demands scores) and a low control score (i.e., lower than the study-specific median of the control scores). This definition of job strain based on the quadrant approach has been widely used and will be the main method to define job strain in the IPD-Work consortium, including the present analyses. However, other approaches to derive measures of job strain from the demands and control scores have also been proposed, including the quotient method (job demands/job control); the logarithmic approach (log[job demands/job control]); and the subtraction approach (job demands minus job control) [38]. As a subsidiary analysis, we evaluated the agreement between the complete and partial job strain scales when applying these alternative job strain definitions, shown in Additional file 2.

### Statistical analysis

The relationship between the complete and partial scales for the demands and control scales was ascertained using Pearson correlation coefficients with accompanying 95% confidence intervals. These were computed using Fisher's transformation. Sensitivity, specificity and Kappa (κ) statistics were calculated to evaluate the agreement between the job strain definitions based on the complete versus partial scales. The following interpretations of the Kappa statistic were utilised [39]: 0.00-0.20 indicates slight agreement, 0.21-0.40 fair, 0.41-0.60 moderate, 0.61-0.80 good/substantial strength of agreement, and 0.81-1.00 a very good/almost perfect agreement. All analyses were performed using SAS version 9.1 (SAS Institute Inc., Cary, NC, USA).

## Results

Pearson's correlation coefficients between the complete demand scale and partial scales are shown in table 3. In all six studies included in the present analysis, the correlation coefficient was r > 0.94 for partial scales comprising four items, and r > 0.90 when the partial scale was based on three items. For partial scales with only two items, the correlation coefficient was somewhat lower (r = 0.76 to 0.88), depending on the cohort and item content.

**Table 3:** Correlation between the complete psychological job demands scale vs. shorter versions of the scale

Job Demands*	Belstress n = 21093	Gazel n = 11365	HeSSup n = 16784	SLOSH n = 10975	WOLF N n = 4704	WOLF S n = 5670
	***r *(95% CI)^†^**	***r *(95% CI)**	***r *(95% CI)**	***r *(95% CI)**	***r *(95% CI)**	***r *(95% CI)**

Complete scale vs. B (4 items)	0.968 (0.967-0.969)	0.951 (0.949-0.953)	0.975 (0.974-0.976)	0.972 (0.970-0.973)	0.969 (0.967-0.971)	0.972 (0.970-0.973)

Complete scale vs. C (4 items)	0.956 (0.954-0.957)	0.943 (0.941-0.945)	0.957 (0.955-0.958)	0.962 (0.960-0.963)	0.954 (0.951-0.956)	0.961 (0.959-0.963)

Complete scale vs. D (3 items)	0.928 (0.927-0.930)	0.903 (0.900-0.907)	0.932 (0.930-0.934)	0.933 (0.931-0.935)	0.927 (0.923-0.931)	0.933 (0.929-0.936)

Complete scale vs. E (3 items)	0.928 (0.926-0.930)	0.902 (0.899-0.906)	0.924 (0.921-0.926)	0.914 (0.911-0.917)	0.914 (0.909-0.919)	0.912 (0.907-0.916)

Complete scale vs. F (2 items)	0.876 (0.872-0.879)	0.854 (0.848-0.858)	0.836 (0.831-0.840)	0.855 (0.850-0.860)	0.837 (0.828-0.845)	0.844 (0.836-0.851)

Complete scale vs. G (2 items)	0.782 (0.776-0.787)	0.759 (0.751-0.767)	0.820 (0.815-0.825)	0.805 (0.799-0.812)	0.808 (0.798-0.818)	0.817 (0.808-0.825)

*Abbreviated items of the complete scale: 1. "Work very fast"; 2. "Work very hard/intensively"; 3. " No excessive work/Too much effort "; 4. "Enough time"; 5. "Conflicting demands".

Version B include items 1, 2, 4, 5; version C items 1, 2, 3, 4; version D items 2, 3, 4; version E items 1, 4, 5; version F items 1, 4; and version G items 4, 5.

^†^Pearson product-moment correlation coefficient (*r*) and 95% confidence interval (95% CI).

Table 4 shows that a largely similar pattern of correlations was observed for the control scale. The correlation coefficients between the complete scale with six items and the partial scales comprising five items were very high (r ≥ 0.96), whereas the relationship between the complete scale and partial scales with two items were slightly lower in magnitude (r = 0.81 to 0.87).

**Table 4:** Correlation between the complete job control scale vs. shorter versions of the scale

Job control*	Belstress n = 21160	Gazel n = 11389	HeSSup n = 16833	SLOSH n = 10981	WOLF N n = 4707	WOLF S n = 5681
	***r *****(95% CI)^†^**	***r *****(95% CI)**	***r *****(95% CI)**	***r *****(95% CI)**	***r *****(95% CI)**	***r *****(95% CI)**

Complete scale vs. B (5 items)	0.975 (0.975-0.976)	0.967 (0.966-0.969)	0.975 (0.974-0.975)	0.977 (0.976-0.977)	0.976 (0.975-0.978)	0.983 (0.982-0.984)

Complete scale vs. C (5 items)	0.974 (0.974-0.975)	0.973 (0.972-0.974)	0.975 (0.975-0.976)	0.959 (0.957-0.960)	0.957 (0.954-0.959)	0.971 (0.969-0.972)

Complete scale vs. D (5 items)	0.974 (0.973-0.975)	0.970 (0.969-0.971)	0.979 (0.978-0.979)	0.981 (0.980-0.982)	0.977 (0.975-0.978)	0.984 (0.984-0.985)

Complete scale vs. E (5 items)	0.967 (0.966-0.968)	0.960 (0.958-0.961)	0.972 (0.971-0.973)	0.960 (0.958-0.961)	0.968 (0.966-0.970)	0.978 (0.977-0.979)

Complete scale vs. F (2 items)	0.839 (0.835-0.843)	0.826 (0.820-0.831)	0.860 (0.856-0.863)	0.814 (0.808-0.821)	0.840 (0.831-0.848)	0.866 (0.860-0.873)

*Abbreviated items of the complete scale: 1. "Learn new things"; 2. "High level of skill"; 3. "Require creativity/initiative"; 4. "Repetitive work"; 5. "A lot of say"/"Deciding what to do"; 6. "Deciding how".

Version B include items: 1,2,4,5,6; version C items: 1,2,3,4,6; version D items:1,3,4,5,6; version E items: 1,2,3,4,5; and version F items: 3,6.

^†^Pearson product-moment correlation coefficient (*r*) and 95% confidence interval (95% CI).

Table 5 presents the sensitivity, specificity and Kappa statistics comparing the job strain definition based on complete and partial job demands and control scales. There was a consistent pattern across the studies, with the agreement between job strain definitions based on complete and partial scales being very good (k > 0.80), and sensitivity > 0.80 in 14 of 18 analyses, when only one item of either job demands scale or job control scale was missing. When the job strain definition was based on three demand items and all six control items, the agreement varied between good and very good (κ > 0.68). This was also seen, with one exception, for job strain definitions based on only two demand items but all six control items. When one or more items were missing in both the demands and control scales, most Kappa statistics (n = 18/24) indicated at least good agreement (κ > 0.60), although for some comparisons (n = 6/24) the agreement was moderate (κ = 0.54 to 0.60). As expected, the sensitivity decreased when several items were missing in one or both scales. The subsidiary analyses using alternative methods to define job strain yielded a similar pattern of results as the main analysis defining job strain by the quadrant approach (Additional file 2, tables 4 and 5).

**Table 5:** The agreement between job strain definitions using the complete vs. partial scales

Job strain	Belstress N = 21024		Gazel N = 11362		HeSSup N = 16773		SLOSH N = 10970		WOLF N N = 4702		WOLF S N = 5667	
Version of partial scales*	**Sensitivity** **Specificity**	**κ**^†^	**Sensitivity** **Specificity**	**κ**	**Sensitivity** **Specificity**	**κ**	**Sensitivity** **Specificity**	**κ**	**Sensitivity** **Specificity**	**κ**	**Sensitivity** **Specificity**	**κ**

**Complete demands and control scale vs**. **complete ****demands and ****partial ****control scale**												

Demands version A, control version C (5 items)	0.85 1.00	0.90	0.87 0.99	0.90	0.86 0.99	0.88	0.92 0.97	0.88	0.97 0.95	0.81	0.89 0.99	0.89

**Complete demands and control scale vs**. **partial ****demands and ****complete ****control scale**												

Demands version B (4 items), control version A	0.83 1.00	0.88	0.77 1.00	0.85	0.93 0.98	0.91	0.97 0.98	0.93	0.74 1.00	0.83	0.98 0.96	0.87

Demands version C (4 items), control version A	0.83 1.00	0.87	0.85 0.99	0.87	0.93 0.97	0.87	0.75 1.00	0.82	0.79 0.99	0.85	0.96 0.96	0.86

Demands version D (3 items), control version A	0.84 0.99	0.86	0.56 1.00	0.68	0.88 0.97	0.85	0.62 1.00	0.72	0.72 0.99	0.79	0.92 0.96	0.84

Demands version E (3 items), control version A	0.90 0.97	0.86	0.81 0.99	0.84	0.91 0.95	0.83	0.62 1.00	0.71	0.76 0.99	0.80	0.61 0.99	0.69

Demands version F (2 items), control version A	0.70 0.99	0.77	0.66 0.99	0.74	0.66 0.98	0.70	0.69 0.98	0.75	0.46 0.99	0.58	0.65 0.97	0.68

**Complete demands and control scale vs**. **partial ****demands and ****partial ****control scale**												

Demands version C (4 items), control version F (2 items)	0.67 0.96	0.67	0.66 0.96	0.64	0.78 0.93	0.68	0.58 0.97	0.62	0.68 0.93	0.58	0.63 0.95	0.62

Demands version E (3 items), control version E (5 items)	0.75 0.97	0.76	0.66 0.99	0.72	0.78 0.95	0.73	0.57 0.98	0.64	0.72 0.97	0.72	0.45 0.99	0.55

Demands version F (2 items), control version D (5 items)	0.65 0.98	0.71	0.66 0.93	0.57	0.62 0.97	0.64	0.63 0.99	0.70	0.45 0.99	0.54	0.57 0.98	0.62

Demands version G (2 items), control version B (5 items)	0.47 0.98	0.55	0.72 0.95	0.66	0.73 0.94	0.67	0.70 0.96	0.70	0.62 0.97	0.64	0.43 0.99	0.54

*Abbreviated items of the complete demands scale (version A): 1. "Work very fast"; 2. "Work very hard/intensively"; 3. "Too much effort/No excessive work"; 4. "Enough time"; 5. "Conflicting demands". Version B include items 1, 2, 4, 5; version C items 1, 2, 3, 4; version D items 2, 3, 4; version E items 1, 4, 5; version F items 1, 4; and version G items 4, 5.

Abbreviated items of the complete control scale (version A): 1. "Learn new things"; 2. "High level of skill"; 3. "Require creativity/initiative"; 4. "Repetitive work"; 5. "A lot of say"/"Deciding what to do"; 6. "Deciding how". Version B include items: 1,2,4,5,6; version C items: 1,2,3,4,6; version D items:1,3,4,5,6; version E items: 1,2,3,4,5; and version F items: 3,6.

^†^Kappa statistic (κ)

## Discussion

The aim of the present analyses was to evaluate the comparability of alternative job demands, job control and job strain measures by assessing agreement against the complete scales. To do so, we analysed data from a total of 70 751 participants in six European cohort studies with complete data on five job demand and six control items. We found very high correlation coefficients between the complete and partial job demands and control scales, which included a minimum of three items. The agreement for the dichotomised job strain measure was 'good' to 'very good' when at least one of the underlying subscales was complete. When one or more of the items of the underlying scales were excluded, this agreement ranged from moderate to good, depending on the specific items left out in the partial versions.

### Strengths and weaknesses

The main strength of the present study is the utilisation of data from multiple independent cohort studies which collectively comprised a very large analytical sample, so providing a high level of statistical precision. Despite slight variation in the exact wording of the questionnaire items between these studies, our findings were remarkably consistent across the six cohort studies drawn from Belgium, France, Finland and Sweden. This supports the generalisability of the present analysis across different settings in four European countries. However, in the studies included in the IPD-Work Consortium, no standardised procedure had been followed to translate the original job strain questionnaire across all studies. Thus, cross-cultural adaptation of the job strain instrument remains a source of error for an individual-participant data meta-analysis project on job strain. Based on a review of job demand and control questions used in 17 European cohort studies, our tests were limited to a total of 10 different partial scales that were available in these studies. It is therefore unclear how other scale modifications, including those with additional study-specific questions, agree with the complete scales. These differences, as well as those related to translation and cultural meaning of the wording, may affect the assessment of demands and control in international comparative studies [40].

### Comparison with previous studies

Previously, a comparison of JCQ and DCQ-like questionnaires has been conducted in 682 participants in the JACE study [20]. The investigators in that study found a moderate agreement between median-based job strain classification using a 14-item JCQ (five demand items, nine control items) compared with the 11-item DCQ (five demand items, six control items). Attempts were made to increase the comparability between the scales by developing comparability-facilitating algorithms, as well as using regression models, in order to convert the DCQ scores to the same scale as the JCQ scores. However, with regard to the median based job strain classification, these transformations did not meaningfully improve the agreement between the JCQ and DCQ [20]. We chose a different approach to harmonise the JCQ and DCQ scales, and used the five and six comparable items in the JCQ and DCQ as the "complete" scales. These provided us a reference measurement to examine the validity of partial versions available in the existing cohort studies.

### Implications

In epidemiological studies, researchers often have to make a trade-off between the amount of data they would like to collect and the amount of data it is possible to collect without increasing the non-response due to overly burdensome inventories. In these circumstances, it is not uncommon for the original scales to be abbreviated. Research using job strain as the exposure measure has not always been based on validated standardised protocols and modifications in the questionnaire may have contributed to inconsistencies in the observed job strain-disease association across various studies. For example, in a recent meta-analysis of ten prospective cohort studies, the authors reported a pooled relative risk of 1.4 (95% confidence intervals 1.1-1.8) for incident coronary heart disease among participants with job strain compared to those without [41]. However, there was significant heterogeneity among datasets, with effect estimates close to the null value, or even opposite to the expected direction, in some studies [41]. Even though our study showed reasonable agreement for all the investigated partial scales with the complete scale, the agreement as well as the sensitivity decreased when several items were missing in the two sub scales of the job strain measure. Lower sensitivity implies increased risk of misclassification of job strain when using abbreviated scales, which may attenuate or inflate a true relationship between job strain and an outcome of interest. When using abbreviated scales the item content is important to consider. The control scale in the job strain index is composed of two dimensions -skill discretion and decision authority- and both these dimensions should be covered in a partial scale to measure the same construct as the complete JCQ/DCQ scale. This was the case in all the 17 studies comprising IPD-Work.

## Conclusions

Information on the agreement between alternative operationalisations of job strain may help with the interpretation of previous findings, and harmonisation of multi-cohort data for pooled analyses. Our study provides information on the agreement between complete and partial job strain scales based on existing data from several European cohort studies. A high agreement for partial scales with at least half of the items of the complete scales, and an accurate classification of job strain when at least one of the scales has no missing items, suggest that these abbreviated scales assess the same underlying concepts as the complete survey instrument. However, all the partial scales in the present study (including the subscales comprising only two items), showed high to reasonable agreement with the complete scales. In order to capture the theoretical background for job strain and to measure the same construct as the complete JCQ/DCQ, it is important that the abbreviated control scales cover both the skill discretion and the decision authority dimensions.

## Competing interests

The authors declare that they have no competing interests.

## Authors' contributions

All authors participated in designing the study, generating hypotheses, interpreting the data and writing or critically reviewing the paper. Eleonor I Fransson, Töres Theorell and Mika Kivimäki analysed the data and wrote the first draft. All authors read and approved the final manuscript.

## Pre-publication history

The pre-publication history for this paper can be accessed here:

http://www.biomedcentral.com/1471-2458/12/62/prepub

## Supplementary Material

Additional file 1 **Description of the studies included in IPD-Work Consortium**. Click here for file

Additional file 2 **Appendix tables **1, 2, 3, 4, 5, **correlations, sensitivity, specificity and Kappa statistics for complete and partial job strain scales defined by the quotient, logarithmic, and subtraction approaches**. Click here for file
