# Supplementary material for: Comparison of alternative versions of the job demand-control scales in 17 European cohort studies: the IPD-Work consortium
Source: BMC Public Health. 2012 Jan 20;12:62. doi: 10.1186/1471-2458-12-62 (PMC3328260; doi:10.1186/1471-2458-12-62)
Supplement: Additional file 2 — Appendix tables 1, 2, 3, 4, 5, correlations, sensitivity, specificity and Kappa statistics for complete and partial job strain scales defined by the quotient, logarithmic, and subtraction approaches. [file 1471-2458-12-62-S2.DOC]

**Appendix 2 Correlations, sensitivity, specificity and Kappa statistics when defining job strain by the quotient, logarithmic and subtraction approaches.**

Job strain given by

the quotient approach:
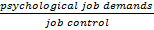


the logarithmic approach:
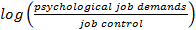


the subtraction approach:
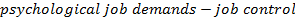


Appendix 2, Table 1. Correlation between the complete job strain scale vs. partial versions of the scale, the quotient approach.

Appendix 2, Table 2. Correlation between the complete job strain scale vs. partial versions of the scale, the logarithmic approach.

Appendix 2, Table 3. Correlation between the complete job strain scale vs. partial versions of the scale, the subtraction approach.

Appendix 2, Table 4. The agreement between job strain definitions using the complete vs. partial scales, the quotient approach.*^†^

Appendix 2, Table 5. The agreement between job strain definitions using the complete vs. partial scales, the subtraction approach. ^†^

*Since the results for the quotient and the logarithmic scales are identical with regards to sensitivity, specificity and kappa statistics, only the table for the quotient approach is included.

^†^ Job strain was defined as values above the 75^th^ percentile on the continuous scale.

**Appendix 2, Table 1. Correlation between the complete job strain scale vs. partial versions of the scale, the quotient approach**

| **Job strain by quotient approach** | **Belstress**  **N=21024** |  | **Gazel**  **N=11362** |  | **HeSSup**  **N=16773** |  | **SLOSH**  **N=10970** |  | **WOLF N**  **N=4702** |  | **WOLF S**  **N=5667** |  |
| --- | --- | --- | --- | --- | --- | --- | --- | --- | --- | --- | --- | --- |
| **Version of partial scales*** | **r (95% CI)^†^** |  | **r (95% CI)** |  | **r (95% CI)** |  | **r (95% CI)** |  | **r (95% CI)** |  | **r (95% CI)** |  |
| **Complete demands and control scale vs. complete demands and partial control scale** | | | | | | | | | | | | |
| Demands version A, control version C (5 items) | 0.982  (0.982-0.983) |  | 0.983  (0.983-0.984) |  | 0.986  (0.985-0.986) |  | 0.980  (0.979-0.981) |  | 0.976  (0.974-0.977) |  | 0.982  (0.981-0.983) |  |
| **Complete demands and control scale vs. partial demands and complete control scale** | | | | | | | | | | | | |
| Demands version B (4 items), control version A | 0.983  (0.983-0.984) |  | 0.968  (0.967-0.970) |  | 0.987  (0.987-0.987) |  | 0.983  (0.983-0.984) |  | 0.979  (0.978-0.980) |  | 0.982  (0.981-0.983) |  |
| Demands version C (4 items), control version A | 0.974  (0.974-0.975) |  | 0.961  (0.960-0.963) |  | 0.977  (0.977-0.978) |  | 0.977  (0.976-0.977) |  | 0.968  (0.967-0.970) |  | 0.973  (0.972-0.975) |  |
| Demands version D (3 items), control version A | 0.957  (0.956-0.959) |  | 0.932  (0.929-0.934) |  | 0.954  (0.952-0.955) |  | 0.950  (0.948-0.952) |  | 0.941  (0.937-0.944) |  | 0.938  (0.935-0.941) |  |
| Demands version E (3 items), control version A | 0.962  (0.961-0.964) |  | 0.944  (0.942-0.946) |  | 0.960  (0.959-0.961) |  | 0.947  (0.945-0.949) |  | 0.939  (0.935-0.942) |  | 0.942  (0.939-0.944) |  |
| Demands version F (2 items), control version A | 0.926  (0.925-0.928) |  | 0.894  (0.890-0.897) |  | 0.915  (0.913-0.918) |  | 0.909  (0.905-0.912) |  | 0.885  (0.879-0.891) |  | 0.896  (0.891-0.901) |  |
| **Complete demands and control scale vs. partial demands and partial control scale** | | | | | | | | | | | | |
| Demands version C (4 items), control version F (2 items) | 0.849  (0.845-0.852) |  | 0.838  (0.833-0.844) |  | 0.862  (0.858-0.866) |  | 0.874  (0.869-0.878) |  | 0.855  (0.847-0.862) |  | 0.859  (0.852-0.866) |  |
| Demands version E (3 items), control version E (5 items) | 0.932  (0.931-0.934) |  | 0.926  (0.923-0.929) |  | 0.937  (0.935-0.938) |  | 0.927  (0.925-0.930) |  | 0.916  (0.911-0.920) |  | 0.927  (0.923-0.931) |  |
| Demands version F (2 items), control version D (5 items) | 0.910  (0.908-0.912) |  | 0.887  (0.883-0.891) |  | 0.909  (0.906-0.912) |  | 0.910  (0.907-0.913) |  | 0.885  (0.878-0.891) |  | 0.890  (0.885-0.895) |  |
| Demands version G (2 items), control version B (5 items) | 0.858  (0.855-0.862) |  | 0.847  (0.842-0.852) |  | 0.855  (0.851-0.859) |  | 0.845  (0.839-0.850) |  | 0.820  (0.811-0.830) |  | 0.842  (0.834-0.849) |  |

*Abbreviated items of the complete demands scale (version A): 1. "Work very fast"; 2. "Work very hard/intensively"; 3. "Too much effort/No excessive work"; 4. "Enough time"; 5. "Conflicting demands". Version B include items 1, 2, 4, 5; version C items 1, 2, 3, 4; version D items 2, 3, 4; version E items 1, 4, 5; version F items 1, 4; and version G items 4, 5. Abbreviated items of the complete control scale (version A): 1. “Learn new things”; 2. “High level of skill”; 3. “Require creativity/initiative”; 4. “Repetitive work”; 5. “A lot of say”/”Deciding what to do”; 6. “Deciding how”. Version B include items: 1,2,4,5,6; version C items: 1,2,3,4,6; version D items:1,3,4,5,6; version E items: 1,2,3,4,5; and version F items: 3,6.

^†^Pearson product-moment correlation coefficient (*r*) and 95% confidence interval (95% CI).

**Appendix 2, Table 2. Correlation between the complete job strain scale vs. partial versions of the scale, the logarithmic approach**

| **Job strain by logarithmic approach** | **Belstress**  **N=21024** |  | **Gazel**  **N=11362** |  | **HeSSup**  **N=16773** |  | **SLOSH**  **N=10970** |  | **WOLF N**  **N=4702** |  | **WOLF S**  **N=5667** |  |
| --- | --- | --- | --- | --- | --- | --- | --- | --- | --- | --- | --- | --- |
| **Version of partial scales*** | **r (95% CI)^†^** |  | **r (95% CI)** |  | **r (95% CI)** |  | **r (95% CI)** |  | **r (95% CI)** |  | **r (95% CI)** |  |
| **Complete demands and control scale vs. complete demands and partial control scale** | | | | | | | | | | | | |
| Demands version A, control version C (5 items) | 0.985  (0.985-0.986) |  | 0.986  (0.986-0.987) |  | 0.989  (0.989-0.990) |  | 0.984  (0.983-0.984) |  | 0.978  (0.977-0.979) |  | 0.983  (0.983-0.984) |  |
| **Complete demands and control scale vs. partial demands and complete control scale** | | | | | | | | | | | | |
| Demands version B (4 items), control version A | 0.979  (0.978-0.980) |  | 0.963  (0.961-0.964) |  | 0.984  (0.984-0.985) |  | 0.979  (0.978-0.980) |  | 0.975  (0.974-0.976) |  | 0.978  (0.977-0.979) |  |
| Demands version C (4 items), control version A | 0.968  (0.967-0.969) |  | 0.957  (0.955-0.958) |  | 0.971  (0.970-0.972) |  | 0.972  (0.971-0.973) |  | 0.964  (0.961-0.965) |  | 0.969  (0.967-0.970) |  |
| Demands version D (3 items), control version A | 0.947  (0.945-0.948) |  | 0.924  (0.921-0.927) |  | 0.938  (0.936-0.940) |  | 0.940  (0.938-0.942) |  | 0.931  (0.927-0.935) |  | 0.928  (0.924-0.931) |  |
| Demands version E (3 items), control version A | 0.955  (0.954-0.956) |  | 0.933  (0.931-0.935) |  | 0.947  (0.945-0.948) |  | 0.938  (0.935-0.940) |  | 0.929  (0.925-0.933) |  | 0.932  (0.928-0.935) |  |
| Demands version F (2 items), control version A | 0.907  (0.905-0.909) |  | 0.875  (0.871-0.879) |  | 0.887  (0.884-0.890) |  | 0.892  (0.888-0.895) |  | 0.868  (0.860-0.875) |  | 0.877  (0.871-0.883) |  |
| **Complete demands and control scale vs. partial demands and partial control scale** | | | | | | | | | | | | |
| Demands version C (4 items), control version F (2 items) | 0.879  (0.875-0.882) |  | 0.866  (0.861-0.870) |  | 0.906  (0.904-0.909) |  | 0.897  (0.894-0.901) |  | 0.883  (0.877-0.889) |  | 0.889  (0.884-0.895) |  |
| Demands version E (3 items), control version E (5 items) | 0.934  (0.933-0.936) |  | 0.918  (0.915-0.920) |  | 0.932  (0.930-0.934) |  | 0.923  (0.920-0.926) |  | 0.911  (0.906-0.916) |  | 0.920  (0.916-0.924) |  |
| Demands version F (2 items), control version D (5 items) | 0.897  (0.894-0.899) |  | 0.871  (0.866-0.875) |  | 0.887  (0.884-0.890) |  | 0.896  (0.892-0.900) |  | 0.870  (0.863-0.877) |  | 0.879  (0.873-0.884) |  |
| Demands version G (2 items), control version B (5 items) | 0.837  (0.833-0.841) |  | 0.827  (0.821-0.833) |  | 0.817  (0.812-0.822) |  | 0.826  (0.820-0.832) |  | 0.802  (0.791-0.812) |  | 0.817  (0.808-0.825) |  |

*Abbreviated items of the complete demands scale (version A): 1. "Work very fast"; 2. "Work very hard/intensively"; 3. "Too much effort/No excessive work"; 4. "Enough time"; 5. "Conflicting demands". Version B include items 1, 2, 4, 5; version C items 1, 2, 3, 4; version D items 2, 3, 4; version E items 1, 4, 5; version F items 1, 4; and version G items 4, 5. Abbreviated items of the complete control scale (version A): 1. “Learn new things”; 2. “High level of skill”; 3. “Require creativity/initiative”; 4. “Repetitive work”; 5. “A lot of say”/”Deciding what to do”; 6. “Deciding how”. Version B include items: 1,2,4,5,6; version C items: 1,2,3,4,6; version D items:1,3,4,5,6; version E items: 1,2,3,4,5; and version F items: 3,6.

^†^Pearson product-moment correlation coefficient (*r*) and 95% confidence interval (95% CI).

**Appendix 2, Table 3. Correlation between the complete job strain scale vs. partial versions of the scale, the subtraction approach**

| **Job strain by subtraction approach** | **Belstress**  **N=21024** |  | **Gazel**  **N=11362** |  | **HeSSup**  **N=16773** |  | **SLOSH**  **N=10970** |  | **WOLF N**  **N=4702** |  | **WOLF S**  **N=5667** |  |
| --- | --- | --- | --- | --- | --- | --- | --- | --- | --- | --- | --- | --- |
| **Version of partial scales*** | **r (95% CI)^†^** |  | **r (95% CI)** |  | **r (95% CI)** |  | **r (95% CI)** |  | **r (95% CI)** |  | **r (95% CI)** |  |
| **Complete demands and control scale vs. complete demands and partial control scale** | | | | | | | | | | | | |
| Demands version A, control version C (5 items) | 0.985  (0.984-0.985) |  | 0.985  (0.985-0.986) |  | 0.988  (0.987-0.988) |  | 0.981  (0.980-0.982) |  | 0.973  (0.971-0.974) |  | 0.980  (0.979-0.981) |  |
| **Complete demands and control scale vs. partial demands and complete control scale** | | | | | | | | | | | | |
| Demands version B (4 items), control version A | 0.980  (0.980-0.981) |  | 0.967  (0.966-0.969) |  | 0.984  (0.984-0.985) |  | 0.983  (0.982-0.983) |  | 0.979  (0.978-0.980) |  | 0.981  (0.980-0.982) |  |
| Demands version C (4 items), control version A | 0.970  (0.969-0.971) |  | 0.957  (0.955-0.958) |  | 0.972  (0.971-0.973) |  | 0.976  (0.975-0.977) |  | 0.968  (0.967-0.970) |  | 0.973  (0.971-0.974) |  |
| Demands version D (3 items), control version A | 0.953  (0.951-0.954) |  | 0.927  (0.924-0.929) |  | 0.949  (0.947-0.950) |  | 0.952  (0.950-0.954) |  | 0.946  (0.943-0.949) |  | 0.944  (0.942-0.947) |  |
| Demands version E (3 items), control version A | 0.958  (0.956-0.959) |  | 0.943  (0.941-0.945) |  | 0.953  (0.952-0.955) |  | 0.948  (0.946-0.949) |  | 0.941  (0.938-0.944) |  | 0.941  (0.938-0.944) |  |
| Demands version F (2 items), control version A | 0.916  (0.914-0.919) |  | 0.887  (0.883-0.891) |  | 0.897  (0.894-0.900) |  | 0.910  (0.907-0.913) |  | 0.888  (0.882-0.894) |  | 0.896  (0.891-0.901) |  |
| **Complete demands and control scale vs. partial demands and partial control scale** | | | | | | | | | | | | |
| Demands version C (4 items), control version F (2 items) | 0.883  (0.880-0.886) |  | 0.865  (0.860-0.870) |  | 0.906  (0.903-0.908) |  | 0.885  (0.881-0.889) |  | 0.869  (0.862-0.876) |  | 0.881  (0.875-0.887) |  |
| Demands version E (3 items), control version E (5 items) | 0.937  (0.935-0.938) |  | 0.926  (0.924-0.929) |  | 0.936  (0.934-0.938) |  | 0.930  (0.928-0.933) |  | 0.922  (0.917-0.926) |  | 0.929  (0.925-0.932) |  |
| Demands version F (2 items), control version D (5 items) | 0.905  (0.903-0.907) |  | 0.880  (0.876-0.884) |  | 0.896  (0.893-0.899) |  | 0.912  (0.909-0.915) |  | 0.888  (0.881-0.894) |  | 0.896  (0.891-0.901) |  |
| Demands version G (2 items), control version B (5 items) | 0.859  (0.855-0.863) |  | 0.857  (0.852-0.861) |  | 0.863  (0.859-0.867) |  | 0.864  (0.859-0.869) |  | 0.848  (0.839-0.856) |  | 0.855  (0.848-0.862) |  |

*Abbreviated items of the complete demands scale (version A): 1. "Work very fast"; 2. "Work very hard/intensively"; 3. "Too much effort/No excessive work"; 4. "Enough time"; 5. "Conflicting demands". Version B include items 1, 2, 4, 5; version C items 1, 2, 3, 4; version D items 2, 3, 4; version E items 1, 4, 5; version F items 1, 4; and version G items 4, 5. Abbreviated items of the complete control scale (version A): 1. “Learn new things”; 2. “High level of skill”; 3. “Require creativity/initiative”; 4. “Repetitive work”; 5. “A lot of say”/”Deciding what to do”; 6. “Deciding how”. Version B include items: 1,2,4,5,6; version C items: 1,2,3,4,6; version D items:1,3,4,5,6; version E items: 1,2,3,4,5; and version F items: 3,6.

^†^Pearson product-moment correlation coefficient (*r*) and 95% confidence interval (95% CI).

**Appendix 2, Table 4. The agreement between job strain definitions using the complete vs. partial scales, the quotient approach**

| **Job strain** | **Belstress**  **N=21024** |  | **Gazel**  **N=11362** |  | **HeSSup**  **N=16773** |  | **SLOSH**  **N=10970** |  | **WOLF N**  **N=4702** |  | **WOLF S**  **N=5667** |  |
| --- | --- | --- | --- | --- | --- | --- | --- | --- | --- | --- | --- | --- |
| **Version of partial scales*** | **Sensitivity**  **Specificity** | **κ**^†^ | **Sensitivity**  **Specificity** | **κ** | **Sensitivity**  **Specificity** | **κ** | **Sensitivity**  **Specificity** | **κ** | **Sensitivity**  **Specificity** | **κ** | **Sensitivity**  **Specificity** | **κ** |
| **Complete demands and control scale vs. complete demands and partial control scale** | | | | | | | | | | | | |
| Demands version A, control version C (5 items) | 0.93  0.98 | 0.90 | 0.74  1.00 | 0.80 | 0.91  0.97 | 0.88 | 0.82  0.99 | 0.86 | 0.63  1.00 | 0.71 | 0.73  0.99 | 0.79 |
| **Complete demands and control scale vs. partial demands and complete control scale** | | | | | | | | | | | | |
| Demands version B (4 items), control version A | 0.90  0.97 | 0.87 | 0.87  0.96 | 0.83 | 0.90  0.97 | 0.88 | 0.87  0.99 | 0.89 | 0.88  0.96 | 0.84 | 0.90  0.98 | 0.89 |
| Demands version C (4 items), control version A | 0.87  0.97 | 0.84 | 0.82  0.97 | 0.82 | 0.87  0.96 | 0.84 | 0.89  0.97 | 0.87 | 0.82  0.98 | 0.82 | 0.89  0.97 | 0.86 |
| Demands version D (3 items), control version A | 0.86  0.95 | 0.81 | 0.79  0.95 | 0.75 | 0.84  0.95 | 0.80 | 0.72  0.99 | 0.77 | 0.78  0.95 | 0.75 | 0.83  0.94 | 0.76 |
| Demands version E (3 items), control version A | 0.82  0.96 | 0.79 | 0.81  0.95 | 0.77 | 0.85  0.95 | 0.80 | 0.84  0.95 | 0.79 | 0.79  0.95 | 0.77 | 0.72  0.98 | 0.75 |
| Demands version F (2 items), control version A | 0.79  0.95 | 0.75 | 0.77  0.93 | 0.71 | 0.77  0.93 | 0.71 | 0.69  0.97 | 0.71 | 0.77  0.94 | 0.71 | 0.67  0.96 | 0.67 |
| **Complete demands and control scale vs. partial demands and partial control scale** | | | | | | | | | | | | |
| Demands version C (4 items), control version F (2 items) | 0.74  0.93 | 0.68 | 0.70  0.93 | 0.64 | 0.76  0.92 | 0.69 | 0.61  0.97 | 0.64 | 0.66  0.95 | 0.65 | 0.76  0.92 | 0.68 |
| Demands version E (3 items), control version E (5 items) | 0.82  0.94 | 0.76 | 0.81  0.94 | 0.75 | 0.82  0.94 | 0.76 | 0.82  0.94 | 0.76 | 0.79  0.94 | 0.75 | 0.79  0.95 | 0.75 |
| Demands version F (2 items), control version D (5 items) | 0.80  0.93 | 0.73 | 0.73  0.94 | 0.69 | 0.78  0.93 | 0.71 | 0.80  0.94 | 0.74 | 0.73  0.94 | 0.70 | 0.77  0.93 | 0.69 |
| Demands version G (2 items), control version B (5 items) | 0.74  0.91 | 0.65 | 0.72  0.91 | 0.63 | 0.73  0.92 | 0.65 | 0.72  0.91 | 0.63 | 0.67  0.92 | 0.61 | 0.71  0.91 | 0.63 |

*Abbreviated items of the complete demands scale (version A): 1. "Work very fast"; 2. "Work very hard/intensively"; 3. "Too much effort/No excessive work"; 4. "Enough time"; 5. "Conflicting demands". Version B include items 1, 2, 4, 5; version C items 1, 2, 3, 4; version D items 2, 3, 4; version E items 1, 4, 5; version F items 1, 4; and version G items 4, 5.

Abbreviated items of the complete control scale (version A): 1. “Learn new things”; 2. “High level of skill”; 3. “Require creativity/initiative”; 4. “Repetitive work”; 5. “A lot of say”/”Deciding what to do”; 6. “Deciding how”. Version B include items: 1,2,4,5,6; version C items: 1,2,3,4,6; version D items:1,3,4,5,6; version E items: 1,2,3,4,5; and version F items: 3,6.

^†^Kappa statistic (κ)

**Appendix 2, Table 5. The agreement between job strain definitions using the complete vs. partial scales, the subtraction approach**

| **Job strain** | **Belstress**  **N=21024** |  | **Gazel**  **N=11362** |  | **HeSSup**  **N=16773** |  | **SLOSH**  **N=10970** |  | **WOLF N**  **N=4702** |  | **WOLF S**  **N=5667** |  |
| --- | --- | --- | --- | --- | --- | --- | --- | --- | --- | --- | --- | --- |
| **Version of partial scales*** | **Sensitivity**  **Specificity** | **κ**^†^ | **Sensitivity**  **Specificity** | **κ** | **Sensitivity**  **Specificity** | **κ** | **Sensitivity**  **Specificity** | **κ** | **Sensitivity**  **Specificity** | **κ** | **Sensitivity**  **Specificity** | **κ** |
| **Complete demands and control scale vs. complete demands and partial control scale** | | | | | | | | | | | | |
| Demands version A, control version C (5 items) | 0.95  0.97 | 0.90 | 0.74  1.00 | 0.80 | 0.83  0.99 | 0.86 | 0.82  0.99 | 0.86 | 0.63  1.00 | 0.71 | 0.73  0.99 | 0.79 |
| **Complete demands and control scale vs. partial demands and complete control scale** | | | | | | | | | | | | |
| Demands version B (4 items), control version A | 0.86  0.98 | 0.86 | 0.87  0.96 | 0.83 | 0.87  0.98 | 0.87 | 0.87  0.99 | 0.89 | 0.89  0.96 | 0.85 | 0.90  0.98 | 0.89 |
| Demands version C (4 items), control version A | 0.87  0.97 | 0.84 | 0.80  0.97 | 0.80 | 0.83  0.97 | 0.83 | 0.84  0.98 | 0.85 | 0.82  0.97 | 0.82 | 0.89  0.97 | 0.86 |
| Demands version D (3 items), control version A | 0.79  0.97 | 0.79 | 0.79  0.94 | 0.75 | 0.79  0.96 | 0.77 | 0.72  0.99 | 0.77 | 0.76  0.97 | 0.77 | 0.83  0.95 | 0.77 |
| Demands version E (3 items), control version A | 0.82  0.96 | 0.79 | 0.81  0.95 | 0.78 | 0.79  0.96 | 0.78 | 0.84  0.95 | 0.80 | 0.79  0.96 | 0.77 | 0.72  0.98 | 0.75 |
| Demands version F (2 items), control version A | 0.80  0.94 | 0.73 | 0.77  0.93 | 0.71 | 0.68  0.96 | 0.68 | 0.69  0.97 | 0.71 | 0.76  0.94 | 0.71 | 0.67  0.96 | 0.67 |
| **Complete demands and control scale vs. partial demands and partial control scale** | | | | | | | | | | | | |
| Demands version C (4 items), control version F (2 items) | 0.66  0.96 | 0.67 | 0.70  0.93 | 0.64 | 0.72  0.95 | 0.69 | 0.61  0.97 | 0.64 | 0.60  0.96 | 0.61 | 0.68  0.95 | 0.66 |
| Demands version E (3 items), control version E (5 items) | 0.83  0.95 | 0.78 | 0.81  0.94 | 0.75 | 0.81  0.94 | 0.76 | 0.82  0.94 | 0.76 | 0.78  0.95 | 0.75 | 0.79  0.95 | 0.75 |
| Demands version F (2 items), control version D (5 items) | 0.80  0.93 | 0.72 | 0.73  0.94 | 0.69 | 0.74  0.94 | 0.69 | 0.80  0.94 | 0.74 | 0.73  0.94 | 0.70 | 0.73  0.94 | 0.68 |
| Demands version G (2 items), control version B (5 items) | 0.70  0.93 | 0.65 | 0.70  0.92 | 0.64 | 0.73  0.92 | 0.66 | 0.67  0.94 | 0.64 | 0.67  0.92 | 0.61 | 0.73  0.91 | 0.64 |

*Abbreviated items of the complete demands scale (version A): 1. "Work very fast"; 2. "Work very hard/intensively"; 3. "Too much effort/No excessive work"; 4. "Enough time"; 5. "Conflicting demands". Version B include items 1, 2, 4, 5; version C items 1, 2, 3, 4; version D items 2, 3, 4; version E items 1, 4, 5; version F items 1, 4; and version G items 4, 5.

Abbreviated items of the complete control scale (version A): 1. “Learn new things”; 2. “High level of skill”; 3. “Require creativity/initiative”; 4. “Repetitive work”; 5. “A lot of say”/”Deciding what to do”; 6. “Deciding how”. Version B include items: 1,2,4,5,6; version C items: 1,2,3,4,6; version D items:1,3,4,5,6; version E items: 1,2,3,4,5; and version F items: 3,6.

^†^Kappa statistic (κ)
